# Investigation of Genetic Structure between Deep and Shallow Populations of the Southern Rock Lobster, *Jasus edwardsii* in Tasmania, Australia

**DOI:** 10.1371/journal.pone.0077978

**Published:** 2013-10-18

**Authors:** Erin M. J. Morgan, Bridget S. Green, Nicholas P. Murphy, Jan M. Strugnell

**Affiliations:** 1 Department of Genetics, La Trobe Institute for Molecular Science, La Trobe University, Bundoora, Australia; 2 Institute for Marine and Antarctic Studies, University of Tasmania, Hobart, Tasmania, Australia; CSIRO, Australia

## Abstract

The southern rock lobster, *Jasus edwardsii*, shows clear phenotypic differences between shallow water (red coloured) and deeper water (pale coloured) individuals. Translocations of individuals from deeper water to shallower waters are currently being trialled as a management strategy to facilitate a phenotypic change from lower value pale colouration, common in deeper waters, to the higher value red colouration found in shallow waters. Although panmixia across the *J. edwardsii* range has been long assumed, it is critical to assess the genetic variability of the species to ensure that the level of population connectivity is appropriately understood and translocations do not have unintended consequences. Eight microsatellite loci were used to investigate genetic differentiation between six sites (three shallow, three deep) across southern Tasmania, Australia, and one from New Zealand. Based on analyses the assumption of panmixia was rejected, revealing small levels of genetic differentiation across southern Tasmania, significant levels of differentiation between Tasmania and New Zealand, and high levels of asymmetric gene flow in an easterly direction from Tasmania into New Zealand. These results suggest that translocation among Tasmanian populations are not likely to be problematic, however, a re-consideration of panmictic stock structure for this species is necessary.

## Introduction

Human-mediated movement of species, known as translocation or assisted migration, is increasing in popularity as a strategy to maintain species abundance, connectivity and diversity. Translocation has been used commonly throughout agricultural history, and it is currently also an important conservation strategy for threatened species [1,2]. Successful translocation of individuals is reliant on a number of biological, behavioural and genetic factors. If translocation programs between populations fail to recognise genetic differences between prospective populations, the process can have serious effects on the species in question, including partial or complete replacement of the local population, competition resulting in population size reduction, inbreeding depression, outbreeding depression and consequent loss in fitness, ‘swamping’ or disease introduction, or loss of localized adaptations [3]. Understanding genetic connectivity between populations is key for effective species management and successful translocations between populations [4]. 

Pilot translocations were trialled in the southern rock lobster (*Jasus edwardsii*), to determine if it was possible to improve value and productivity of the Australian stock [5,6]. Between 2004 and 2008, 30,000 lobsters were translocated from deeper water (>60 metres depth) locations in Tasmania, Australia, and released in shallower water locations (0-30 m depth) [5,7]. Importantly, there are clear phenotypic differences between these shallow and deep water populations of southern rock lobster [6]. The shallow water phenotype is characterised by a darker red shell colour, larger body size and shape, higher vitality for live transport and faster growth rate, as compared to the deep water phenotype [6,8,9]. These have been identified as a large red morph and a small pale morph [5]. These pilot translocations were a biological proof of concept experiment, and following the success of this study (translocated individuals changed phenotype) a larger scale of translocation of 100,000 individuals from deep to shallow water is underway to determine if it may be commercially viable [5]. 

Phenotypic differences between shallow and deep populations of the southern rock lobster are due to differences in habitat, and are not genetic, as pilot studies have shown that translocated, pale individuals change to the more desired phenotype after a single moult [5,8]. Although the phenotypic differences are understood to be plastic it is nevertheless possible that genetic structuring exists between shallow water and deeper water populations of lobsters (despite their long pelagic larval duration of ~2 years) which is driven by other factors. For example, in a recent study of *Panulirus interruptus* (California spiny lobster) Iacchei et al [10] show slight but significant population differentiation between sites shown to be driven by higher proportions of kin present within sites than would be expected by chance. *P. interruptus* also possesses a long larval duration (240-330 days) [10]. Indeed there is a growing body of evidence suggesting that most marine populations are not genetically homogenous across broad scales [4,10-14].

Like many marine species with long larval phases, the southern rock lobster has long been assumed to be panmictic throughout the range of Australasia [15-17]. Knowledge of genetic stock structure is based upon a single genetic study of nucleotide sequence polymorphisms in the mitochondrial genome [15] and a few allozyme studies [16,17]. However, recent research has demonstrated significant population subdivision and dispersal patterns in the southern rock lobster around New Zealand [18], countering these assumptions of panmixia. Additionally, larval transport models via ocean currents also suggest that population structure in southern rock lobster is likely to be complex [19,20]. Microsatellite markers are highly variable neutral markers widely accepted as being unaffected by selection. They are therefore a useful tool to resolve the level of population structure and gene flow in a species assumed to have phenotypic plasticity and high connectivity. Microsatellite markers were recently developed for the southern rock lobster [21], and with higher statistical power in resolving a finer level of population structure in highly connected marine species such as this, we can now evaluate population connectivity for this species at a level appropriate to identify genetic structure.

This study aims to use microsatellite markers to investigate genetic differentiation among Tasmanian populations of the southern rock lobster, where translocations are under consideration as an ongoing management strategy. Analysis of genetic structure is evaluated at different levels, including between 1) shallow and deep water populations, 2) fine scale geographic separation of Tasmanian populations and 3) the oceanic divide of Tasmania and a New Zealand site. This will help to determine if current translocation efforts stand to negatively impact the southern rock lobster, and if there is significance in the scale and directionality of connectivity for this species. Potential patterns in connectivity and source-sink recruitment relationships may be important in the appropriate management and success of translocation for this species in the future.

## Materials and Methods

### Sample Collection

**Figure 1:**
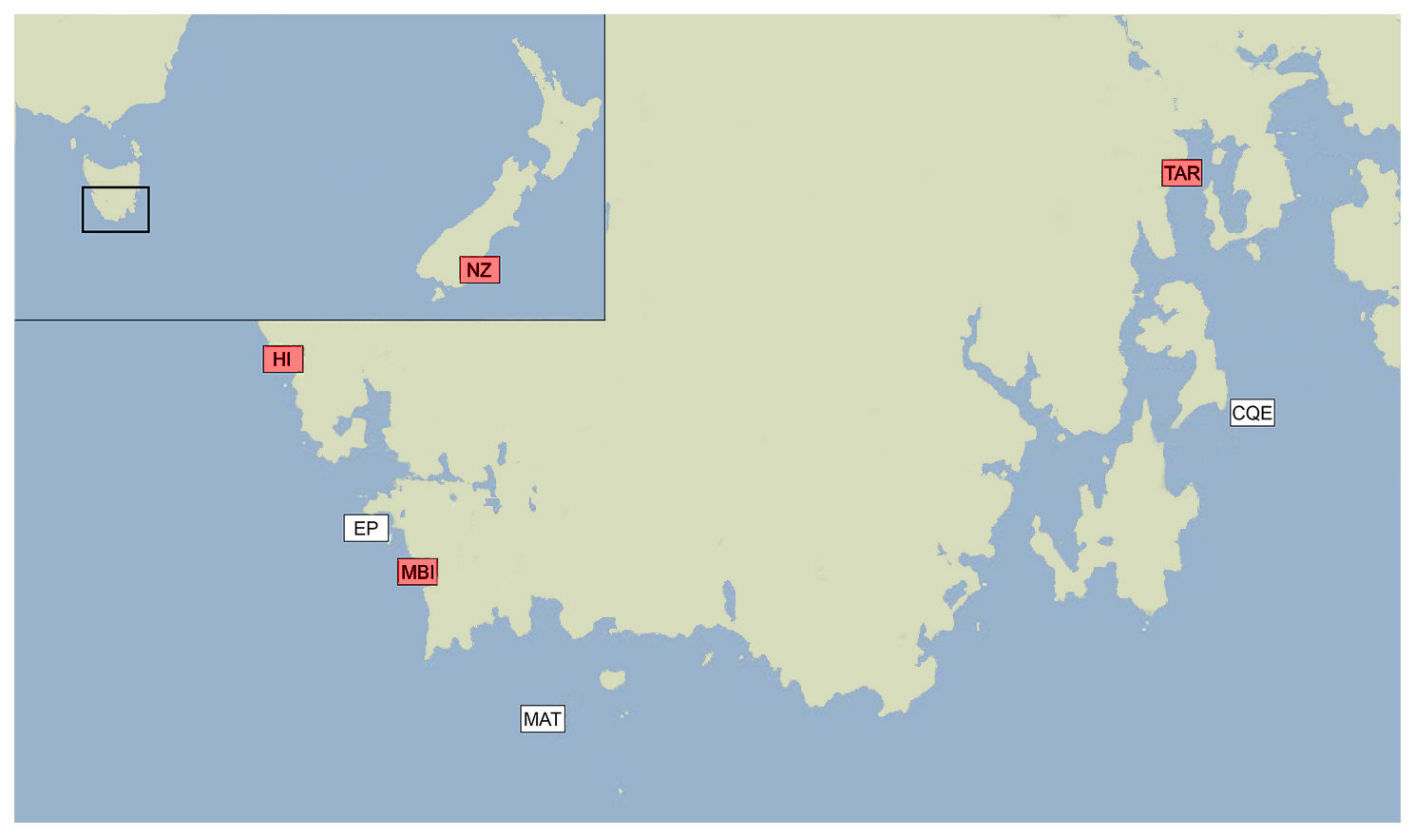
Map of sample sites of southern rock lobsters across the southern coast of Tasmania, Australia. Red squares indicate shallow water sites of HI, Hobbs Island; MBI, Mutton Bird Island; TAR, Taroona Reserve; white squares indicates deep water sites of EP, East Pyramids; MAT, Maatsyuker Island; CQE, Cape Queen Elizabeth. Inset (top left) depicts sampling location in NZ, New Zealand; ~2,000 km from Tasmanian sites.

Lobsters were sampled from six sites across the southern coast of Tasmania, and one site from New Zealand (NZ) (Figure 1). All handling of rock lobsters in this study met the ABS/ASAB guidelines for ethical treatment of animals. Tasmanian samples were collected in State-regulated waters where no permission is required to sample lobsters, and one site (Taroona Reserve) which is a research reserve, closed to other forms of fishing. Permission was obtained through a special research permit provided to IMAS for the purpose of rock lobster research. The permit to sample lobsters for scientific research in Taroona reserve was issued by the wild fisheries branch of the Department of Primary Industries and Water, under the living marine resources management act 1995, section 4. Permit number 12098, permit Holder Dr Caleb Gardner IMAS University of Tasmania. No specific permit was required for the other 5 sites as they were in state waters where fishing for rock lobster is allowed.

Baited traps deployed and collected from research and commercial vessels were used to catch lobsters. All handling of Tasmanian rock lobsters in this study met the Australian Government National Health and Medical Research Council code of practice for the care and use of animals for scientific purposes. Although currently ethics approval is not required for research on invertebrates under this code of practice, the guidelines for ethical and humane treatment of animals in research were followed in all handling of lobsters. Sampling of rock lobster did not involve interactions with endangered or threatened species. Three shallow water sites sampled (Taroona Reserve [TAR], Mutton Bird Island [MBI, South of Port Davey] and Hobbs Island [HI, North of Port Davey]), were between 0 and 30 metres water depth, and comprised of lobsters with red coloured phenotypes. Three deep water sites sampled (Maatsyuker Island [MAT, tagged and translocated into Taroona Reserve between 2004 and 2008], Cape Queen Elizabeth [CQE] and East Pyramids [EP, Port Davey]) were greater than 60 metres in depth, and were largely populated by pale coloured lobsters. Distances between sample sites (by sea) range from 10 km (between EP and MBI) to 220 km apart (between HI and TAR). We considered the effects of geographic and oceanic distance, between shallow and deep populations and any potentially resulting genetic patterns on subsequent translocations within the stock.

Samples of rock lobster in Taroona reserve were collected on 1-4 February 2012 (including translocated individuals from Maatsyuker Island). A clip of tissue from the pleopod was stored in 95% ethanol, and the lobster released. Pleopod tissue samples from other Tasmanian sites were collected from 15 January to 15 February 2012. In addition, tissue samples were taken from lobsters collected from Taieri Mouth, Otago Harbour and Moeraki on the south island of New Zealand (Figure 1) during August 2011. No specific permissions were required for the NZ lobsters as they were harvested by commercial fishermen in compliance with standard NZ Ministry of Fisheries regulations and as a commercially harvested species they are neither endangered nor protected. All pleopod samples were assigned unique ID tags and stored individually in 95% ethanol at -20°C.

### DNA Extraction, PCR Amplification and Genotyping

DNA was extracted from a total of 460 individuals using the high salt extraction method [22]. Nine microsatellite loci identified by Thomas and Bell [21] for use on *J. edwardsii* (Table 1), were assigned unique fluorophores (FAM, NED, VIC, PET) [23], for fluorescent tagging of DNA in a PCR reaction. PCR reactions were performed to amplify selected DNA fragments with MyTaq RedMix (Bioline) in 11µl PCR reaction mixtures using the PCR protocol recommended by Thomas and Bell [21]. Each mix contained 5.43µl of MyTaq RedMix, 0.07µl of 10mM forward primer, 0.22µl of 10mM reverse primer, 0.17µl of 5pmol/µl fluorophore label, 4.11µl of H_2_O and 1µl of concentrated DNA product from sample extractions [21,23]. 

**Table 1:** Microsatellite loci characteristics modified from Thomas and Bell [19].

Locus	GenBank Accession Number	Repeat motif	Ta	Size_M_ (bp)
JE_01	JN806248	(CA)_62_	70–60	121-261
JE_17	JN806249	(ATAC)_13_	70–60	165-253
JE_NS	JN806252	(CAG)_50_	70–60	286-553
JE_JM	JN806253	(TTAGG)_3_ (TA)_2_ (GGTTA)_25_	70–60	190-389
JE_05	JN806254	(TACCT)_20_	70–60	Na
JE_LZ	JN806255	(GGTTA)_33_	70–60	263-568
JE_40	JN806250	(GTAG)_62_	60–50	357-509
JE_07	JN806251	(CGT)_52_	60–50	398-465
JE_9M	JN806256	(ACCTA)_9_ (ACCAA)_3_ (ACCTA)_7_	60–50	187-322

Ta, Touchdown PCR protocol annealing temperature; (Size_M_), modified base pair range from Thomas and Bell estimates [19].

PCR products were sent to the Australian Genome Research Facility Ltd (AGRF) for capillary separation. Results were carefully scrutinised by eye using GENEIOUS PRO version 5.6.4 [24], using the microsatellite analysis external plug-in [25]. PCRs were repeated for those individuals for which unclear or missing signals were obtained for up to 3 more times before being classed as missing data (and scored as 0, 0).

### Genetic Polymorphism

Binned genotypes scored were formatted in GENALEX version 6.4 [26]. MICRO-CHECKER version 2.2.3 [27], was used to check allelic data for negative, zero or out of range values. Null allele frequencies were estimated in FREENA [28]. Due to a significant portion of null alleles found (>10% at any locus) in FREENA, false homozygote frequencies were used to adjust the number of null alleles by re-naming potential nulls as 999 [29]. Further analysis of data used both the adjusted allele frequency data and raw data to assess the effect of null alleles on results. GENEPOP version 4.1.3 [30,31], FSTAT version 2.9.3 [32], and GENALEX were used to analyse basic descriptive statistics within and between populations. Allelic diversity, observed versus expected levels of heterozygosity and levels of inbreeding (using the Fixation index estimate) were calculated in GENALEX. FSTAT was used to calculate allelic richness. GENEPOP was used to test for significant departures from Hardy Weinberg Equilibrium. The number of private alleles for each population and linkage disequilibrium between loci were assessed using GENEPOP.

### Genetic Connectivity and Population Subdivision

Pairwise F-statistics (Fst's) were calculated in FSTAT between assigned groups of individuals. Fst's were tested by hierarchical comparisons between: 1) all populations, 2) shallow water and deep water groups and 3) paired groups of Tasmania and New Zealand.

### Population Structure

STRUCTURE version 2.3.4 [33], was used to cluster individuals. The admixture model was used to assume some level of connection between populations. A burn-in length of 100,000, 500,000 MCMC replicates, 3 iterations and a search for the number of clusters (K) between 1 and 10 (the assumed number of populations present plus 3) were used. STRUCTURE HARVESTER online version 0.6.92 [34], was used to evaluate results using the Evanno method [35], and DISTRUCT version 1.1 [36] used to graphically display results. Discriminant analysis of principle components (DAPC) [37], was used to assess data using the program R version 2.15.1 [38], run via R STUDIO version 0.96.331 [39]. PCA was performed in R using ADEGENET version 1.3-4 [40,41]. 60 principle components were retained as predictors for discriminant analysis.

### Migration and Directionality of Gene Flow

BAYESASS version 3.0.1 [42] was used to assess admixture [43]. Raw genotype data was converted for input analysis into BAYESASS using FORMATOMATIC version 0.8.1 [44]. Trace output convergence was assessed using TRACER version 1.5 [45]. 21,000,000 iterations and 5,000,000 burn in length were used to produce convergent trace outputs. The data was tested in a hierarchical manner between different geographic distances.

## Results

### DNA Extraction, PCR Amplification and Genotyping

A total of 460 individuals were genotyped for eight microsatellite loci. Despite numerous attempts to optimise PCR conditions for all nine microsatellite loci from Thomas and Bell [21], locus JE_05 was successful in less than 10% of reactions, and so was excluded from this study. Of the eight remaining microsatellite loci, less than three percent of genotypes were unable to be scored during analysis. Genotype data is available from Dryad Digital Repository (http://doi.org/10.5061/dryad.656gf). 

### Genetic Polymorphism

A significant frequency of null alleles were detected from loci JE_01, JE_LZ, JE_17, JE_40 and JE_07 using MICRO-CHECKER [27], although null alleles averaged no more than 11 percent for each locus (across all populations) (Table S1). The exception to this was locus JE_01, which had an average of 22 percent null alleles (Table S1). Of these loci, where a significant frequency of null alleles was detected, 3 were suggested to have 'possible stuttering', most likely due to null allele effects (JE_01, JE_17 and JE_07) [27]. Null allele frequencies for these five loci were quantified by the EM algorithm [46] (Table S1), and adjusted using FREENA [28], to correct for a homozygote excess by random re-labelling of homozygote null alleles with the unique number 999, using estimates of false homozygote frequencies. No large allele dropout was detected, and loci JE_NS, JE_9M and JE_JM had non-significant (less than 10%) null alleles. All loci were found to be in linkage equilibrium. 

Allelic richness for each population was similar (~17-18 alleles) (Table 2). TAR, HI and NZ populations have a lower number of private alleles (6-9), compared with EP, which had a slightly higher number of private alleles (19). 

**Table 2:** Descriptive statistics across all populations.

Population:	N	N_A_	N_PA_	AR	H_O_	H_E_	F_IS_
TAR	98	29.875	9	18.095	0.924	0.908	-0.018
MBI	68	28.000	12	17.805	0.921	0.907	-0.014
HI	59	25.750	6	17.720	0.913	0.906	-0.007
MAT	73	28.500	10	18.245	0.926	0.905	-0.022
CQE	67	27.875	11	18.058	0.922	0.903	-0.021
EP	70	29.250	19	18.351	0.928	0.911	-0.018
NZ	25	17.125	6	16.542	0.866	0.846	-0.032

N, number of individuals per population; N_A_, average number of alleles across all loci per population; N_PA_, average number of private alleles across all loci per population; AR, average allelic richness across all loci per population; H_O_, observed level of heterozygosity; H_E_, expected level of heterozygosity; F_IS_, Fixation index (inbreeding coefficient). TAR, Taroona Reserve; MBI, Mutton Bird Island; HI, Hobbs Island; MAT, Maatsyuker Island; CQE, Cape Queen Elizabeth; EP, East Pyramids; NZ, New Zealand.

### Genetic Connectivity and Population Subdivision

F-statistics were used to compare across 1) all populations, 2) solely between Tasmanian populations and 3) between red (shallow) and pale (deep) populations of Tasmania. After Bonferroni correction, the data set indicated a significant difference between NZ and the six Tasmanian populations, the largest oceanographic distance compared (Fst=0.0290-0.0342) (Table 3). Fst analysis of the data set indicated a p<0.05 significant difference between shallow and deep populations of MBI and CQE (Fst=0.0021), between the shallow populations of HI and MBI (Fst=0.001) and between the deep population of MAT and the shallow population HI (Fst=0.003). The dataset was also analysed using a “leave-one-out” approach for loci JE_07, JE_01 and JE_17 and without any of these markers, due to an unusual repeat motif in the former, and the presence of null alleles and potential stuttering in all three (Tables S2-S2.3). Excluding these loci from analysis altered some Fst relationships between each of the populations, as would be expected when any contributing loci were removed from an analysis. However, significant differences after Bonferroni corrections were still evident between Tasmanian populations (both individually and grouped) and New Zealand.

**Table 3:** F-statistics across all populations.

	TAR	MBI	HI	MAT	CQE	EP
MBI	0.0002					
HI	0.0005	**0.0010**				
MAT	0.0016	0.0024	**0.0030**			
CQE	0.0003	**0.0021**	0.0015	-0.0004		
EP	0.0012	0.0026	-0.0005	0.0008	0.0002	
NZ	0.0292*	0.0320*	0.0342*	0.0342*	0.0290*	0.0312*

Data set of Fst values, bold indicates significant values of p value <0.05, * significant values after Bonferroni correction of p<0.002381. TAR, Taroona Reserve; MBI, Mutton Bird Island; HI, Hobbs Island; MAT, Maatsyuker Island; CQE, Cape Queen Elizabeth; EP, East Pyramids; NZ, New Zealand.

When Tasmanian populations were combined and compared to the NZ population in a pairwise Fst test, analysis showed significant levels of differentiation (Fst=0.0305) over this large distance. When populations from Tasmania only were compared by grouping the three shallow populations and the three deep populations in a pairwise analysis, no significant difference was detected. Overall, F-statistics indicated a significant difference between Tasmanian and New Zealand individuals, and a small yet still significant level of differentiation among some populations of Tasmania, yet no consistent differences between shallow and deep populations.

### Population Structure

**Figure 2:**
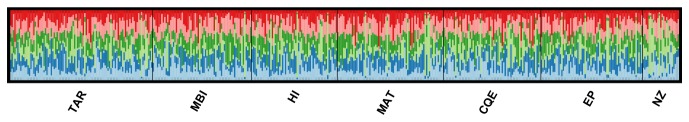
STRUCTURE assignment of individuals across all populations into clusters of best fit at K=6. Colours indicate percentage contribution of individuals to assigned clusters (y axis), individuals represented by each line (x axis), black lines separate populations from which individuals belong. TAR, Taroona Reserve; MBI, Mutton Bird Island; HI, Hobbs Island; MAT, Maatsyuker Island; CQE, Cape Queen Elizabeth; EP, East Pyramids; NZ, New Zealand.

An analysis of clusters in STRUCTURE revealed no clear grouping of individuals sampled (Figure 2). Grouping the six populations around Tasmania and the one population in New Zealand suggested a K of best fit as six clusters, however, no clear assignment of individuals to singular clusters was visualised. The site of New Zealand showed individuals with a very minor difference to the remaining grouping of Tasmanian sites, with a slightly larger contribution of individuals to cluster three (light green, ~13% greater than the average for Tasmanian sites), and a lower contribution to clusters two (dark blue, ~5% less) and four (dark green, ~5% less), potentially suggesting small differences in genetic character between New Zealand and Tasmanian populations (Table 4). Other comparisons showed no more than a maximum of four percent difference between the proportion of any one site assigned to a cluster, and most averaged only a one percent difference. A hierarchical subdivision in STRUCTURE was created from individuals of Tasmania, with New Zealand removed to reveal potential substructure on a finer scale. STRUCTURE determined a best fit of five population clusters (K = 5), however, no clear assignment of individuals to any clusters was evident (data not shown). A structure plot of k = 2 genetic clusters (Figure S1), showed no detectable difference between New Zealand and Tasmanian populations. This indicated that any differences in the k = 6 plot were not significant enough to be identified using a more simple comparison. Overall, STRUCTURE results indicated no clear genetic clusters that could be associated with depth or small scale connectivity, but rather indicated small but insignificant differences between the larger geographic distances between Tasmania and New Zealand.

**Table 4:** Percentage contribution of each population to assigned clusters (K=6) using STRUCTURE.

Population:	Contribution to Clusters (Percentage):					
	1	2	3	4	5	6
TAR	17	17	16	16	17	17
MBI	19	15	13	19	19	15
HI	16	18	16	17	17	16
MAT	16	17	16	18	17	17
CQE	18	16	18	17	16	16
EP	15	19	16	17	17	17
NZ	18	12	29	12	17	13

TAR, Taroona Reserve; MBI, Mutton Bird Island; HI, Hobbs Island; MAT, Maatsyuker Island; CQE, Cape Queen Elizabeth; EP, East Pyramids; NZ, New Zealand

**Figure 3:**
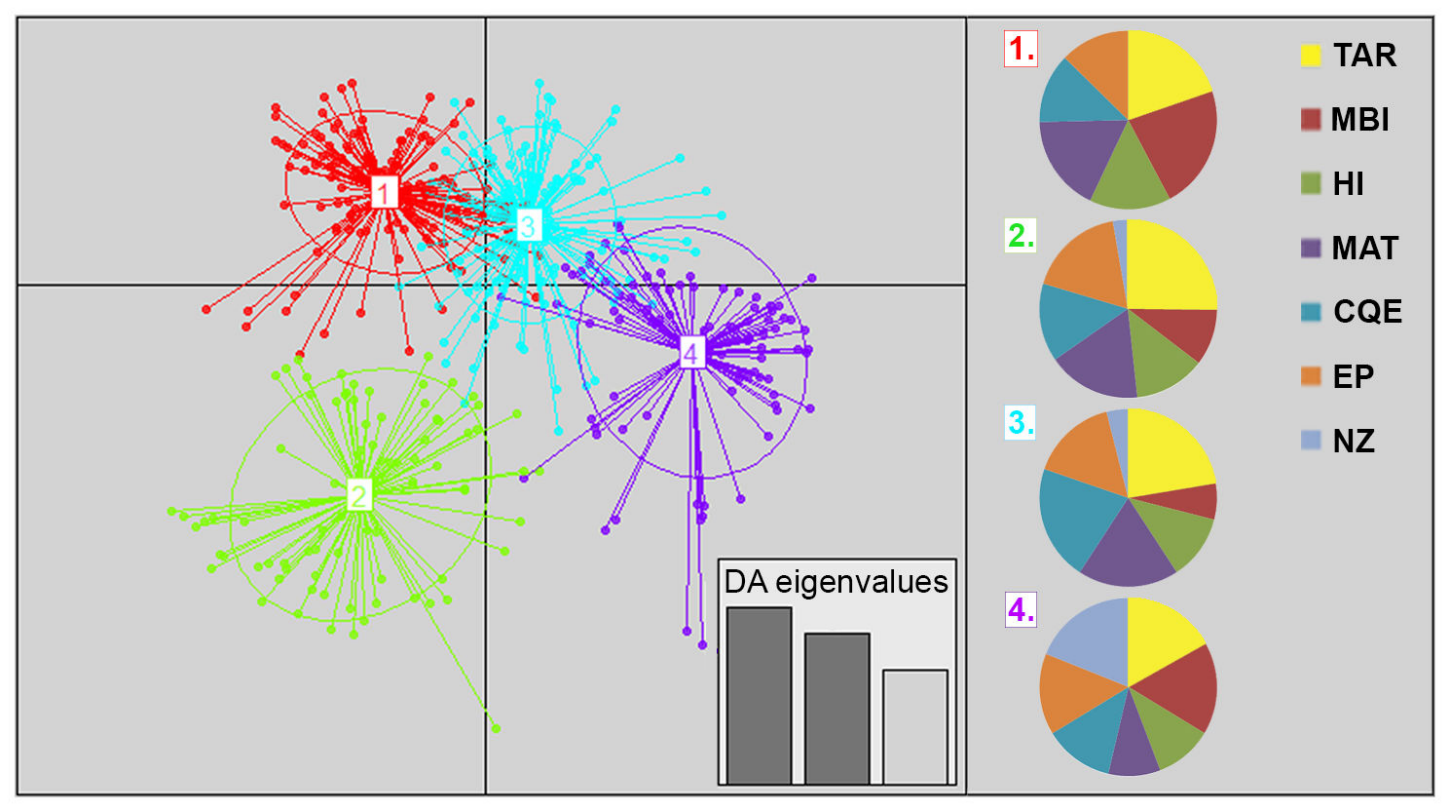
DAPC assignment and subsequent grouping of individuals with optimum clusters of K=4. Plot of DAPC for four assigned genetic clusters, each indicated by different colours. Dots represent different individuals, bottom right inset shows eigenvalues of principle components in relative magnitude. Pie graphs on right indicate the number value of individuals from each population (TAR, MBI, HI, MAT, CQE, EP, NZ) assigned to the relative genetic clusters created by DAPC.

DAPC was tested on all individuals with a best fit for clusters found at K = 4 (Figure 3). The majority of individuals across the Tasmanian sample sites were assigned to genetically distinct clusters of 1 and 2 (58-69%), with a lesser contribution to clusters 3 and 4 (31-42%) (Table 5). Some number of individuals from each of the Tasmanian sample sites belonged to each of the clusters. The New Zealand population had the majority of individuals (72%) assigned to cluster 4, with less contribution to clusters 2 and 3 and no individuals assigned to cluster 1 (indicating this cluster as unique to Tasmania) (Table 5). A hierarchical analysis from DAPC, removing the New Zealand population to refine Tasmanian populations substructure, again clustered individuals into a K = 4 grouping, with no population structure evident among the Tasmanian sites.

**Table 5:** Percentage contribution of each population to the clusters assigned by DAPC.

Population:	Contribution to Clusters (Percentage):			
	1	2	3	4
TAR	29	38	17	16
MBI	47	22	7	24
HI	36	32	15	17
MAT	34	34	19	12
CQE	27	31	24	18
EP	26	37	17	20
NZ	0	16	12	72

TAR, Taroona Reserve; MBI, Mutton Bird Island; HI, Hobbs Island; MAT, Maatsyuker Island; CQE, Cape Queen Elizabeth; EP, East Pyramids; NZ, New Zealand

Analysis using STRUCTURE and DAPC suggested some level of differentiation present between individuals from Tasmania compared to New Zealand, however, no fine scale structuring was noted amongst populations of Tasmania, which suggested a high level of admixture between populations.

### Migration and Directionality of Gene Flow

Evaluation of migrants or admixture between populations was analysed using BAYESASS on hierarchical levels of 1) between all populations and 2) between combined populations of Tasmania and New Zealand. BAYESASS permits migration rates to be asymmetric but they must be small, the number of migrants per generation must not exceed a third, and in scenarios with low genetic differentiation (Fst<0.02) the program will struggle to define resulting migration patterns [43]. A pairwise comparison of each of the six Tasmanian populations and the New Zealand population therefore struggled to define levels of migration between Tasmanian populations. This was because Tasmania potentially had more than one third of migrations per generation, and Fst values between populations of Tasmania were noted as low (0.002). BAYESASS failed to distinguish which populations across Tasmania were exchanging an accurate number of migrants, as significant levels of migration in any one population changed between other Tasmanian populations each time the test was replicated (Table S3). Importantly, migration levels between the six Tasmanian populations and New Zealand were always consistent, despite the inconsistent results observed amongst Tasmanian populations (Table S3). A comparison between grouped populations of Tasmania and the New Zealand population (Table 6) was more consistent, as Fst values between the two populations were adequate (~0.03), migration levels were thought to be less than a third, and decreasing the number of populations increases the accuracy of estimations of migration rates [43]. This comparison suggested that 32% of New Zealand individuals sampled were migrants from Tasmania, whereas less than 1% of Tasmanian individuals were from New Zealand (Table 6). 

**Table 6:** Migration rates (posterior probabilities) between Tasmania and New Zealand.

	TAS	NZ
TAS	***0.9987*** (0.0012)	0.0013 (0.0012)
NZ	0.3208 (0.0121)	***0.6792*** (0.0121)

Bold/italicised values indicate self recruitment, values in parentheses indicate standard deviation, left column indicates where migrants travelled to, top row indicates where migrants originated from. TAS, Tasmania; NZ, New Zealand.

To take into consideration any effects of unequal number in sample sizes, pairwise comparisons of each individual Tasmanian population were run against the New Zealand population. Results showed no differences except that New Zealand was shown to realistically contribute closer to 1-3% of migrants to Tasmania (Table S4). BAYESASS indicated that although migration rates were high amongst Tasmanian populations, they were lower between Tasmania and New Zealand, and in the order of 10 to 30 times more frequent from Tasmania to New Zealand than in the reverse direction.

## Discussion

### Genetic Viability of Translocation in Tasmania

Pilot scale translocations of lobsters from deep to shallow waters around the southern coast of Tasmania and Southern Australia were financially and biologically beneficial [5,7,8,47]. This current study suggests that the translocation of lobsters collected from deep water locations and released in shallow water around southern Tasmania is not inadvertently mixing genetically distinct populations. With no significant genetic differences between the shallow (red phenotypes) and the deep (pale) lobster populations, and a high level of migration and subsequent gene flow between all Tasmanian populations, translocation of lobsters in southern Tasmania is unlikely to lead to any genetic mixing of differently adapted gene pools.

There is minor evidence of some population structure, with low, yet statistically significant individual pairwise comparisons between some Tasmanian sites. With Fst values <0.003, it is probable that these values are not biologically significant [48,49], however, a number of marine species have weaker values of genetic differentiation between populations that are still highly biologically significant and likely to represent important levels of unique stock structure [50,51]. Therefore low levels of statistically significant structure in *J. edwardsii* should not be disregarded completely. Rather, more complete sampling across the Tasmanian coast and Australia in general is required for a more definitive conclusion on genetic stocks. Detailed studies of population structure have not yet investigated patterns of genetic structure across Australia. The need for further study on Tasmanian populations, and the genetic stock structure of *J. edwardsii* across Australia is emphasised by the recent study of New Zealand populations [18]. Thomas (2010) determined that *J. edwardsii* was not homogeneous throughout its range in New Zealand, and rejected the null hypothesis of panmixia [18], although, like the present study, Thomas’s conclusions are based on small, yet significant population differences.

Nevertheless, finding statistically significant differences in pairwise comparisons of populations is not sufficient enough to confidently conclude that such populations are demonstrating an important level of genetic sub-structuring [50]. Statistical power will be high when using multiple and highly variable markers such as microsatellites on a large dataset such as this [48,52]. Therefore, small levels of difference in allele frequencies that are potentially unrelated to the true population structure (and hence not meaningful on a biological level) can be presented as statistically significant [48,52]. For this reason, assumptions about what is biologically meaningful genetic differentiation should be interpreted with a degree of care [48]. What is truly decided as meaningful should be interpreted with an understanding of the biological question in mind [50], as well as with a number of different statistical methods and an understanding of the limitations of each. In an evaluation of potential genetic differences between shallow and deep water populations across southern Tasmania, no tests supported any significant genetic differences between them, hence, it can be understood that small levels of differentiation noted between these individual locations is not due to differences in depth, and translocation from deep water into shallow is likely to have no negative consequences for these populations.

### Evidence for Large Scale Population Subdivision

Assumptions of population panmixia between Australia and New Zealand [15] appear to be incorrect. Significant genetic structure is evident between Tasmania and New Zealand. This is similar to that found by Thomas [18] in a comparison of a South Australian population and New Zealand. These results are in contrast to previous assumptions of a mixed New Zealand and Australian stock of *J. edwardsii* that are supported by models of larval trajectories that suggest trans-Tasman dispersal from Australia to New Zealand [19]. The understanding about population connectivity in lobsters between Australia and New Zealand populations has changed over time. These two populations were historically thought of as separate species, *J. edwardsii* and *J. novaehollandiae* (based on minor differences in morphology) [53], until electrophoretic analysis concluded that the level of differentiation was like that of different populations, not species, and the two populations formed one stock [17]. As some level of gene flow was evident, with supporting evidence from biological, biochemical, oceanography reports, life history characteristics and mtDNA analysis [15,16], the two species became known as one continuous population. The results of the present study, whilst not predicting a return to a separate species status, suggest the two countries do not share a single population.

This study provides a detailed investigation of the populations of southern Tasmania, some of which are currently involved in translocation projects. Clearly though it represents only a preliminary study of *J. edwardsii* across its entire range of southern Australia and New Zealand. There is evidence that gene flow between distant populations does not occur equally in both directions; with both the results of this study and those of Thomas 2012 [18], suggesting a significantly larger number of migrants travelling to New Zealand, than from New Zealand in the opposite direction. This suggests Australia is a potential source of new migrants and subsequent gene flow into New Zealand, acting as a source of stock recruitment. Clearly more populations are needed to be included to determine the full extent of asymmetric gene flow, not only across the Tasman Sea but also along the coast of Australia. If these results stand up in further study, then the New Zealand populations may be dependent on the supply of Australian genetic material.

Given the significant influence ocean currents have been suggested to have on population differentiation between Tasmania and New Zealand, they may also play a significant role in determining population connectivity amongst Australian populations. Migration rates were unable to be appropriately resolved between the sampled populations across Tasmania, clearly indicating important gaps in sampling that could have led to a determination of the level of self recruitment, or source stocks for southern Tasmania. Hydrological and gene flow modelling suggests a dominant eastward flow of the transport of larvae between populations [20], which for Tasmania to New Zealand, results support [18,19]. Details of localised patterns in ocean eddies, currents and associated depths, strengths and directions, are not well studied enough to understand patterns in local source-sink relationships on a fine a scale as that across any one (or two) management zones (like that of sites across Southern Tasmania).

There are a number of large currents across the expanse of southern Australasia that suggest source-sink relationships and an easterly pattern of step wise recruitment driving gene flow in this species [20]. Given that the results presented here suggest a source-sink relationship between Australia and New Zealand (respectively), a larger scale study is required to confirm the influence of ocean currents on population structure. For example the most westerly (Western Australia) and northerly (New South Wales) limits of the range of this species may serve as important source populations for those in South Eastern Australia and New Zealand and therefore these areas should be targeted in future studies. If an eastern flow in stock source recruits throughout the range of the southern Australian coast is confirmed, this may have an important effect on the viability of translocations between populations. Over-exploitation of a source population may therefore have a serious effect not on the stock exploited but on the population to the east which may rely on this stock for recruits.

## Conclusions

This analysis did not identify any scale of population structure that would suggest any genetic differences between shallow (red) and deep (pale) populations. There is a significant level of genetic differentiation between Tasmania and New Zealand, and therefore the assumption of widespread population panmixia can be rejected. Although large scale translocations are genetically viable in this region of Tasmania, it is important to understand that if the indications of asymmetric gene flow and population differentiation found are transferable across the rest of this species range, then translocations should only be undertaken on a local scale. Similarly, finding significant genetic structure in an important fishery species, where previously none had been identified, means a much more detailed assessment of lobster connectivity across the range may find more unique genetic stocks, and important source sink relationships which will have important implications for successful translocations and stock structure management schemes. Given these findings, further research in this area is essential, as current management of the southern rock lobster fishery reflects national and state boundaries rather than the species biology.

## Supporting Information

Figure S1 **STRUCTURE assignment of individuals across all populations into clusters of K=2.** Colours indicate percentage contribution of individuals to assigned clusters (y axis), individuals represented by each line (x axis), black lines separate populations from which individuals belong. TAR, Taroona Reserve; MBI, Mutton Bird Island; HI, Hobbs Island; MAT, Maatsyuker Island; CQE, Cape Queen Elizabeth; EP, East Pyramids; NZ, New Zealand.
(TIF)

Table S1 **Estimates of null allele frequencies across all populations by loci.** Significant frequencies of greater than 10 percent null alleles indicated in bold. TAR, Taroona Reserve; MBI, Mutton Bird Island; HI, Hobbs Island; MAT, Maatsyuker Island; CQE, Cape Queen Elizabeth; EP, East Pyramids; NZ, New Zealand.
(DOCX)

Table S2 **Fst's across all populations without locus JE_07.** Data set of Fst values. Bold indicates significant values of p value <0.05, * indicates significant values after Bonferroni correction of p<0.002381. TAR, Taroona Reserve; MBI, Mutton Bird Island; HI, Hobbs Island; MAT, Maatsyuker Island; CQE, Cape Queen Elizabeth; EP, East Pyramids; NZ, New Zealand.
(Table S2.1) Fst's across all populations without locus JE_17.
Data set of Fst values. Bold indicates significant values of p value <0.05, * indicates significant values after Bonferroni correction of p<0.002381. TAR, Taroona Reserve; MBI, Mutton Bird Island; HI, Hobbs Island; MAT, Maatsyuker Island; CQE, Cape Queen Elizabeth; EP, East Pyramids; NZ, New Zealand.
(Table S2.2) Fst's across All Populations without Locus JE_01
Data set of Fst values. Bold indicates significant values of p value <0.05, * indicates significant values after Bonferroni correction of p<0.002381. TAR, Taroona Reserve; MBI, Mutton Bird Island; HI, Hobbs Island; MAT, Maatsyuker Island; CQE, Cape Queen Elizabeth; EP, East Pyramids; NZ, New Zealand.
(Table S2.3) Fst's across all populations without loci JE_07, _17 and _01
Data set of Fst values. Bold indicates significant values of p value <0.05, * indicates significant values after Bonferroni correction of p<0.002381. TAR, Taroona Reserve; MBI, Mutton Bird Island; HI, Hobbs Island; MAT, Maatsyuker Island; CQE, Cape Queen Elizabeth; EP, East Pyramids; NZ, New Zealand.
(DOCX)

Table S3 **Migration rates (posterior probabilities) across all populations.** Bold/italicised values indicate self recruitment, left column indicates where migrants travelled to, top row indicates where migrants originated from. TAR, Taroona Reserve; MBI, Mutton Bird Island; HI, Hobbs Island; MAT, Maatsyuker Island; CQE, Cape Queen Elizabeth; EP, East Pyramids; NZ, New Zealand.
(DOCX)

Table S4 **Migration rates (posterior probabilities) compared between each Tasmanian population and New Zealand.** Bold/italicised values indicate self recruitment, values in parentheses indicate standard deviation, left column indicates where migrants travelled to, top row indicates where migrants originated from. TAR, Taroona Reserve; MBI, Mutton Bird Island; HI, Hobbs Island; MAT, Maatsyuker Island; CQE, Cape Queen Elizabeth; EP, East Pyramids; NZ, New Zealand
(DOCX)
