# Supplementary figures and images for: Investigation of Genetic Structure between Deep and Shallow Populations of the Southern Rock Lobster, Jasus edwardsii in Tasmania, Australia
Source: PLoS One. 2013 Oct 18;8(10):e77978. doi: 10.1371/journal.pone.0077978 (PMC3820960; doi:10.1371/journal.pone.0077978)

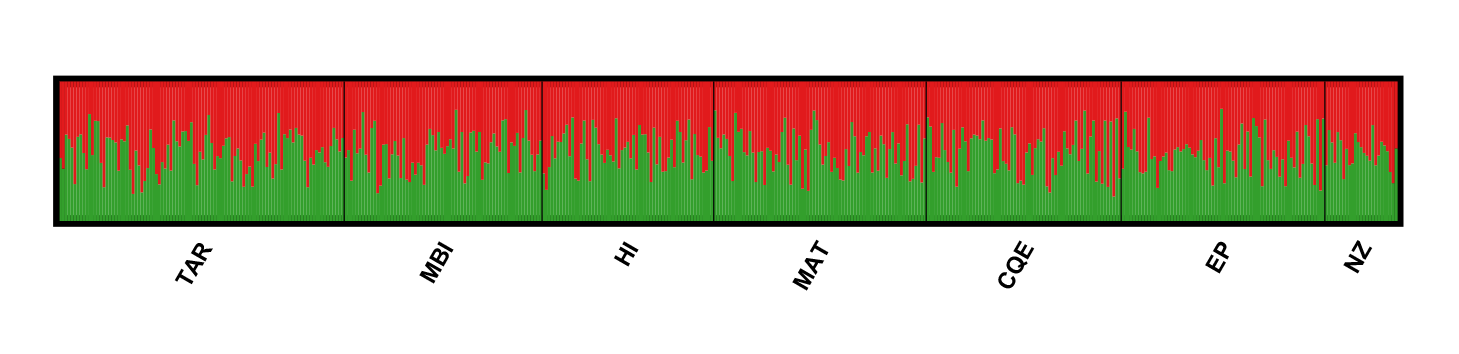

Supplement: Figure S1 — STRUCTURE assignment of individuals across all populations into clusters of K=2. Colours indicate percentage contribution of individuals to assigned clusters (y axis), individuals represented by each line (x axis), black lines separate populations from which individuals belong. TAR, Taroona Reserve; MBI, Mutton Bird Island; HI, Hobbs Island; MAT, Maatsyuker Island; CQE, Cape Queen Elizabeth; EP, East Pyramids; NZ, New Zealand. (TIF) [file pone.0077978.s001.tif]
