# Supplementary material for: Investigation of Genetic Structure between Deep and Shallow Populations of the Southern Rock Lobster, Jasus edwardsii in Tasmania, Australia
Source: PLoS One. 2013 Oct 18;8(10):e77978. doi: 10.1371/journal.pone.0077978 (PMC3820960; doi:10.1371/journal.pone.0077978)
Supplement: Table S1 — Estimates of null allele frequencies across all populations by loci. Significant frequencies of greater than 10 percent null alleles indicated in bold. TAR, Taroona Reserve; MBI, Mutton Bird Island; HI, Hobbs Island; MAT, Maatsyuker Island; CQE, Cape Queen Elizabeth; EP, East Pyramids; NZ, New Zealand. (DOCX) [file pone.0077978.s002.docx]

**Table S1. Estimates of null allele frequencies across all populations by loci**

| Population | Loci | | | | | | | |
| --- | --- | --- | --- | --- | --- | --- | --- | --- |
|  | JE_01 | JE_LZ | JE_17 | JE_NS | JE_9M | JE_40 | JE_JM | JE_07 |
| TAR | **0.201** | 0.064 | **0.105** | 0.021 | 0.025 | **0.101** | 0.045 | **0.167** |
| MBI | **0.172** | **0.123** | 0.076 | 0.035 | 0.001 | 0.073 | 0.022 | **0.137** |
| HI | **0.227** | **0.118** | 0.067 | 0.006 | 0.008 | 0.046 | 0.015 | **0.160** |
| MAT | **0.242** | **0.171** | **0.112** | 0.025 | 0.013 | **0.136** | 0.067 | 0.092 |
| CQE | **0.267** | 0.082 | 0.077 | 0.000 | 0.000 | 0.096 | 0.074 | 0.073 |
| EP | **0.234** | 0.088 | 0.089 | 0.020 | 0.053 | 0.085 | 0.055 | **0.111** |
| NZ | **0.213** | 0.036 | 0.084 | 0.015 | 0.000 | 0.062 | 0.028 | 0.055 |

Significant frequencies of greater than 10 percent null alleles indicated in bold. TAR, Taroona Reserve; MBI, Mutton Bird Island; HI, Hobbs Island; MAT, Maatsyuker Island; CQE, Cape Queen Elizabeth; EP, East Pyramids; NZ, New Zealand.
