# Supplementary material for: Investigation of Genetic Structure between Deep and Shallow Populations of the Southern Rock Lobster, Jasus edwardsii in Tasmania, Australia
Source: PLoS One. 2013 Oct 18;8(10):e77978. doi: 10.1371/journal.pone.0077978 (PMC3820960; doi:10.1371/journal.pone.0077978)
Supplement: Table S2 — Fst's across all populations without locus JE_07. Data set of Fst values. Bold indicates significant values of p value <0.05, * indicates significant values after Bonferroni correction of p<0.002381. TAR, Taroona Reserve; MBI, Mutton Bird Island; HI, Hobbs Island; MAT, Maatsyuker Island; CQE, Cape Queen Elizabeth; EP, East Pyramids; NZ, New Zealand. (Table S2.1) Fst's across all populations without locus JE_17. Data set of Fst values. Bold indicates significant values of p value <0.05, * indicates significant values after Bonferroni correction of p<0.002381. TAR, Taroona Reserve; MBI, Mutton Bird Island; HI, Hobbs Island; MAT, Maatsyuker Island; CQE, Cape Queen Elizabeth; EP, East Pyramids; NZ, New Zealand. (Table S2.2) Fst's across All Populations without Locus JE_01 Data set of Fst values. Bold indicates significant values of p value <0.05, * indicates significant values after Bonferroni correction of p<0.002381. TAR, Taroona Reserve; MBI, Mutton Bird Island; HI, Hobbs Island; MAT, Maatsyuker Island; CQE, Cape Queen Elizabeth; EP, East Pyramids; NZ, New Zealand. (Table S2.3) Fst's across all populations without loci JE_07, _17 and _01 Data set of Fst values. Bold indicates significant values of p value <0.05, * indicates significant values after Bonferroni correction of p<0.002381. TAR, Taroona Reserve; MBI, Mutton Bird Island; HI, Hobbs Island; MAT, Maatsyuker Island; CQE, Cape Queen Elizabeth; EP, East Pyramids; NZ, New Zealand. (DOCX) [file pone.0077978.s003.docx]

**Table S2. Fst's across all populations without locus JE_07**

|  | TAR | MBI | HI | MAT | CQE | EP |
| --- | --- | --- | --- | --- | --- | --- |
| MBI | **0.0009** |  |  |  |  |  |
| HI | 0.0009 | **0.0011** |  |  |  |  |
| MAT | 0.0012 | 0.0022 | **0.0027** |  |  |  |
| CQE | 0.0000 | **0.0024*** | 0.0009 | 0.0002 |  |  |
| EP | 0.0007 | 0.0023 | -0.0013 | 0.0018 | 0.0006 |  |
| NZ | 0.0006 | **0.0050** | **0.0039** | **0.0048** | **0.0029*** | 0.0022 |

Data set of Fst values. Bold indicates significant values of p value <0.05, * indicates significant values after Bonferroni correction of p<0.002381. TAR, Taroona Reserve; MBI, Mutton Bird Island; HI, Hobbs Island; MAT, Maatsyuker Island; CQE, Cape Queen Elizabeth; EP, East Pyramids; NZ, New Zealand.

During allele scoring it was clear that microsatellite loci JE_07 could not be scored into bins of a three base pair repeat motif as suggested by Thomas & Bell, 2011. After examination of the eight microsatellite sequences (from GenBank), JE_07 was noted as having a sequence that did not follow the suggested three base pair repeat motif. Therefore JE_07 was binned into single repeat units to ensure all genotypes could be appropriately scored. F statistics tests carried out excluded JE_07 to evaluate the potential effect this microsatellite may have on overall results, and although it did slightly alter some estimates of Fst's (Table S2) (as would be expected if any contributing microsatellite was excluded from a pair wise analysis), it did not change the overall significance of data produced.

When re-evaluating the data set without the locus JE_07 (that was identified with unusual mutations during initial analysis), the significance of some pair wise relationships changed (Table S2). However, the overall significance of the level of differentiation between NZ and most Tasmanian populations (MBI, HI, MAT and CQE) was still present, and when pooled Tasmanian populations were compared to geographically isolated NZ, the dataset still reported a significant difference (Fst=0.0027). Analysis of the dataset without JE_07 suggested a significant comparison after Bonferroni correction (Fst=0.0024) between deep population CQE and shallow population MBI. The data set additionally suggested a p<0.05 significance between shallow populations of TAR and MBI (Fst=0.0009), and west coast comparisons of shallow populations of HI and MBI (Fst=0.0011) and the deep population of MAT and with shallow population HI (Fst=0.0027) (Table S2).

**Table S2.1. Fst's across all populations without locus JE_17**

|  | TAR | MBI | HI | MAT | CQE | EP |
| --- | --- | --- | --- | --- | --- | --- |
| MBI | 0.0006 |  |  |  |  |  |
| HI | 0.0011 | **0.0017** |  |  |  |  |
| MAT | 0.0015 | 0.0019 | **0.003** |  |  |  |
| CQE | 0.0005 | **0.0027*** | **0.0024** | -0.0004 |  |  |
| EP | 0.0012 | 0.0032 | -0.0001 | 0.0006 | 0.0008 |  |
| NZ | **0.0339*** | **0.0372*** | **0.0400*** | **0.0391*** | **0.0336*** | **0.0361*** |

Data set of Fst values. Bold indicates significant values of p value <0.05, * indicates significant values after Bonferroni correction of p<0.002381. TAR, Taroona Reserve; MBI, Mutton Bird Island; HI, Hobbs Island; MAT, Maatsyuker Island; CQE, Cape Queen Elizabeth; EP, East Pyramids; NZ, New Zealand.

**Table S2.2. Fst's across all populations without locus JE_01**

|  | TAR | MBI | HI | MAT | CQE | EP |
| --- | --- | --- | --- | --- | --- | --- |
| MBI | -0.0004 |  |  |  |  |  |
| HI | 0.0050 | **0.0012** |  |  |  |  |
| MAT | 0.0020 | 0.0023 | **0.0036*** |  |  |  |
| CQE | 0.0000 | **0.0015** | **0.0021** | -0.0003 |  |  |
| EP | 0.0013 | 0.0027 | -0.0002 | 0.0008 | 0.0006 |  |
| NZ | **0.0340*** | **0.036*** | **0.0386*** | **0.0390*** | **0.0324*** | **0.0354*** |

Data set of Fst values. Bold indicates significant values of p value <0.05, * indicates significant values after Bonferroni correction of p<0.002381. TAR, Taroona Reserve; MBI, Mutton Bird Island; HI, Hobbs Island; MAT, Maatsyuker Island; CQE, Cape Queen Elizabeth; EP, East Pyramids; NZ, New Zealand.

**Table S2.3. Fst's across all populations without loci JE_07, _17 and _01**

|  | TAR | MBI | HI | MAT | CQE | EP |
| --- | --- | --- | --- | --- | --- | --- |
| MBI | 0.0009 |  |  |  |  |  |
| HI | 0.0019 | **0.0023** |  |  |  |  |
| MAT | 0.0014 | 0.0014 | **0.0036*** |  |  |  |
| CQE | -0.0001 | **0.0024*** | **0.0029** | 0.0007 |  |  |
| EP | 0.0007 | 0.0033 | -0.0006 | 0.0018 | 0.0020 |  |
| NZ | 0.0029 | 0.0071 | **0.0065** | **0.0071*** | **0.0039** | **0.0035** |

Data set of Fst values. Bold indicates significant values of p value <0.05, * indicates significant values after Bonferroni correction of p<0.002381. TAR, Taroona Reserve; MBI, Mutton Bird Island; HI, Hobbs Island; MAT, Maatsyuker Island; CQE, Cape Queen Elizabeth; EP, East Pyramids; NZ, New Zealand.

Tables S2-S2.2 show the Fst values and p values when comparing across all populations without each of the three loci (JE_07, JE_17 and JE_01) that were identified as having both null alleles and potential stuttering due to null alleles. Additionally, table S2.3 shows these values when all three loci are removed at the same time. As mentioned when discussing JE_07 above, some relationships between populations did change, as would be expected when removing any contributing loci in a dataset. However, the overall significance found when comparing Tasmania to New Zealand did not change. When removing each of the three loci and all three loci at once, significant differences were still noted between Tasmania and New Zealand (Fst values of 0.0027*, 0.0346**, 0.0352** and 0.0044** for loci JE_07, _01, _17 and for the three loci combined being removed respectively).
