# Supplementary material for: Investigation of Genetic Structure between Deep and Shallow Populations of the Southern Rock Lobster, Jasus edwardsii in Tasmania, Australia
Source: PLoS One. 2013 Oct 18;8(10):e77978. doi: 10.1371/journal.pone.0077978 (PMC3820960; doi:10.1371/journal.pone.0077978)
Supplement: Table S3 — Migration rates (posterior probabilities) across all populations. Bold/italicised values indicate self recruitment, left column indicates where migrants travelled to, top row indicates where migrants originated from. TAR, Taroona Reserve; MBI, Mutton Bird Island; HI, Hobbs Island; MAT, Maatsyuker Island; CQE, Cape Queen Elizabeth; EP, East Pyramids; NZ, New Zealand. (DOCX) [file pone.0077978.s004.docx]

**Table S3: Migration rates (posterior probabilities) across all populations**

|  | TAR | MBI | HI | MAT | CQE | EP | NZ |
| --- | --- | --- | --- | --- | --- | --- | --- |
| TAR | ***0.6704*** | 0.0033 | 0.0033 | 0.0033 | 0.3133 | 0.0033 | 0.0032 |
| MBI | 0.0042 | ***0.6713*** | 0.0042 | 0.0042 | 0.3077 | 0.0042 | 0.0042 |
| HI | 0.0047 | 0.0047 | ***0.6719*** | 0.0047 | 0.3045 | 0.0047 | 0.0047 |
| MAT | 0.0042 | 0.0041 | 0.0041 | ***0.6713*** | 0.3082 | 0.0041 | 0.0041 |
| CQE | 0.0065 | 0.0064 | 0.0064 | 0.0060 | ***0.9617*** | 0.0070 | 0.0061 |
| EP | 0.0041 | 0.0041 | 0.0041 | 0.0041 | 0.3084 | ***0.6711*** | 0.0041 |
| NZ | 0.0090 | 0.0090 | 0.0091 | 0.0089 | 0.2775 | 0.0090 | ***0.6776*** |

Bold/italicised values indicate self recruitment, left column indicates where migrants travelled to, top row indicates where migrants originated from. TAR, Taroona Reserve; MBI, Mutton Bird Island; HI, Hobbs Island; MAT, Maatsyuker Island; CQE, Cape Queen Elizabeth; EP, East Pyramids; NZ, New Zealand.
