# Supplementary material for: Investigation of Genetic Structure between Deep and Shallow Populations of the Southern Rock Lobster, Jasus edwardsii in Tasmania, Australia
Source: PLoS One. 2013 Oct 18;8(10):e77978. doi: 10.1371/journal.pone.0077978 (PMC3820960; doi:10.1371/journal.pone.0077978)
Supplement: Table S4 — Migration rates (posterior probabilities) compared between each Tasmanian population and New Zealand. Bold/italicised values indicate self recruitment, values in parentheses indicate standard deviation, left column indicates where migrants travelled to, top row indicates where migrants originated from. TAR, Taroona Reserve; MBI, Mutton Bird Island; HI, Hobbs Island; MAT, Maatsyuker Island; CQE, Cape Queen Elizabeth; EP, East Pyramids; NZ, New Zealand (DOCX) [file pone.0077978.s005.docx]

**Table S4. Migration rates (posterior probabilities) compared between each Tasmanian population and New Zealand**

|  | TAR | NZ |
| --- | --- | --- |
| TAR | ***0.9772*** (0.0140) | 0.0228 (0.0140) |
| NZ | 0.3133 (0.0194) | ***0.6867*** (0.0194) |
|  | MBI | NZ |
| MBI | ***0.9863*** (0.0114) | 0.0137 (0.0114) |
| NZ | 0.3024 (0.0217) | ***0.6976*** (0.0217) |
|  | HI | NZ |
| HI | ***0.9641*** (0.0242) | 0.0359 (0.0242) |
| NZ | 0.2988 (0.0240) | ***0.7012*** (0.0240) |
|  | MAT | NZ |
| MAT | ***0.9893*** (0.0090) | 0.0107 (0.0090) |
| NZ | 0.3112 (0.0224) | ***0.6888*** (0.0224) |
|  | CQE | NZ |
| CQE | ***0.9627*** (0.0188) | 0.0373 (0.0188) |
| NZ | 0.2992 (0.0261) | ***0.7008*** (0.0261) |
|  | EP | NZ |
| EP | ***0.9705*** (0.0164) | 0.0295 (0.0164) |
| NZ | 0.3015 (0.0222) | ***0.6985*** (0.0222) |

Bold/italicised values indicate self recruitment, values in parentheses indicate standard deviation, left column indicates where migrants travelled to, top row indicates where migrants originated from. TAR, Taroona Reserve; MBI, Mutton Bird Island; HI, Hobbs Island; MAT, Maatsyuker Island; CQE, Cape Queen Elizabeth; EP, East Pyramids; NZ, New Zealand
